# Comparative Physicochemical and Catalytic Study of Nanocrystalline Mg-Al Hydrotalcites Precipitated with Inorganic and Organic Bases

**DOI:** 10.3390/nano12162775

**Published:** 2022-08-13

**Authors:** Robert Karcz, Bogna D. Napruszewska, Anna Walczyk, Joanna Kryściak-Czerwenka, Dorota Duraczyńska, Wojciech Płaziński, Ewa M. Serwicka

**Affiliations:** Jerzy Haber Institute of Catalysis and Surface Chemistry, Polish Academy of Sciences, Niezapominajek 8, 30-239 Krakow, Poland

**Keywords:** hydrotalcite, alkali-free synthesis, crystallinity, organic base, tetrabutylammonium hydroxide, choline hydroxide, starch, biotemplate, Baeyer–Villiger oxidation

## Abstract

Synthetic Mg-Al hydrotalcites (HT) are environmentally friendly solid bases frequently applied as catalysts in base catalyzed reactions. The most common synthesis method, using NaOH as precipitant, is problematized by the possibility of introducing undesired Na contamination. Alkali-free synthesis is usually performed with NH_3_aq, a precipitant which is less efficient in incorporation of Mg into HT lattice. In the present work, organic bases, tetrabutylammonium hydroxide and choline hydroxide, were successfully employed as precipitating agents in a new alkali-free route of Mg-Al HT synthesis. HT solids were also obtained with inorganic bases, NH_3_aq and NaOH. Characterization with X-ray diffraction, elemental analysis, scanning electron microscopy, Fourier-transform infrared spectroscopy and thermogravimetry/differential scanning calorimetry, confirmed the formation of nanocrystalline HT compounds with all employed bases. HT prepared with NH_3_aq exhibited an Mg deficit, which was detrimental to the catalytic activity in base catalyzed reactions. The effect was attributed to the tendency of Mg^2+^ to form ammine complexes, a conclusion supported by quantum mechanical calculations. HT prepared with NaOH showed the highest crystallinity, which was unfavorable for catalytic application. The addition of starch to the synthesis medium provided a means by which to diminish the crystal size of all HT precipitates. Catalytic tests of the Baeyer–Villiger oxidation of cyclohexanone demonstrated that the highest yields of ε-caprolactone were obtained with fine-crystalline HT catalysts prepared with organic bases in the presence of a starch template.

## 1. Introduction

**Figure 1:**
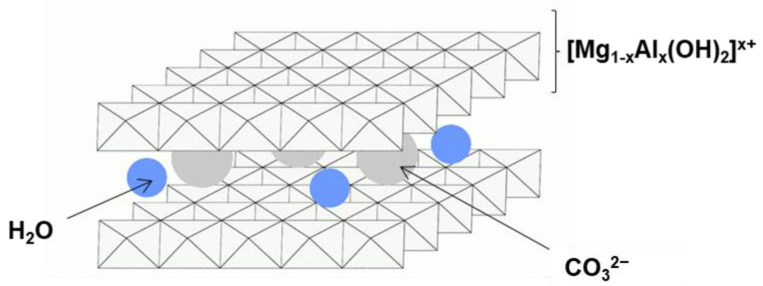
Schematic illustration of HT structure.

Hydrotalcite (HT) is a naturally occurring magnesium hydroxycarbonate mineral, with a formula of Mg_6_Al_2_(OH)_16_CO_3_∙4H_2_O. HT is alternatively referred to as anionic clay or layered double hydroxide. It is built of brucite-like Mg(OH)_2_ layers in which part of Mg^2+^ is replaced by Al^3+^. The excess positive layer charge generated by substitution is compensated for by incorporation of hydrated carbonate anions into the interlayer space [1]. Mg-Al compounds with an HT structure can be easily prepared in the laboratory. The general formula of synthesized solids is usually expressed in the brucite-related form [Mg^2+^_1–x_Al^3+^_x_(OH)_2_] ^x+^(CO_3_)^2−^_x/2_·nH_2_O. Their structure is schematically shown in Figure 1.

It is generally assumed that the value of x enabling the preparation of pure HT phase ranges, approximately, from 0.2 to 0.4 [2,3], although there are reports on the synthesis of magnesium-rich or aluminium-rich HT compounds with compositions outside this limit [4,5,6]. HTs are environmentally friendly solids with anion exchange properties and the basicity is easily tunable by making an adjustment of the Mg/Al ratio. As a result, synthetic Mg-Al HTs (as received or thermally decomposed to mixed oxides) have numerous applications as catalysts, catalyst supports, sorbents, anion exchangers, plastic fillers, hosts for bioactive molecules, etc. [2,3,7,8,9,10]. The HT structure is very flexible and can accommodate a number of M^2+^ and M^3+^ cations other than Mg^2+^ and Al^3+^. For this reason, HTs are excellent precursors for multimetallic mixed oxide systems [2,3,7,11].

The most frequently used method of HT preparation is batch co-precipitation, which involves the reaction of a solution containing an appropriate proportion of Mg and Al salts with solutions of alkali hydroxide and carbonate. Typically, NaOH and Na_2_CO_3_ are applied as co-precipitating agents [2,3,7,12,13,14], but their use involves the possibility of HT contamination with sodium, which is a drawback in the case of HT synthesis for catalytic applications. The leaching of residual Na in the liquid reaction medium may affect the reaction product and/or result in an unstable catalytic performance, as the initial effect of promoting the catalyst basicity fades with time [15,16,17]. Na impurity is also undesired in mixed oxide catalysts derived from transition metal containing HT-like compounds, used in processes requiring a combination of redox and acid sites. There, the Na residues may adversely affect the catalyst performance by decreasing its reducibility, causing sintering of the active phase, blocking the acid function, or generating a coke deposit [18,19,20,21]. It is possible to significantly reduce the level of sodium content by prolonged washing, but even then a certain degree of Na contamination remains [22,23,24,25]. The problem can be resolved by employing precipitants which do not contain sodium, e.g., ammonia or urea [2,3,4,5,9,12,13,14,15,26,27,28,29]. However, the application of both reactants has limitations in the case of HT-like solids with metal cations capable of the formation of ammine complexes. In such a case, the HT phase may not form or the product may show a deficit of the complex-forming cation [12,14,16,28,29,30,31,32].

Our interest in synthesizing alkali-free nanocrystalline HT materials has been prompted by recent experience with the preparation of fine crystalline HT solids for catalytic applications by carrying out HT synthesis in the presence of a starch template [32,33]. A number of studies have shown that a decrease in Mg-Al HT crystal size is beneficial for catalytic performance in base catalyzed reactions [24,34,35,36,37,38,39]. Our idea of using starch solution as medium for HT precipitation proved to be a simple and effective means of reducing the crystal size of HT material. Starch can be easily removed by calcination, hence the absence of alkali contaminants allows for the preparation of mixed oxide catalysts/adsorbents with the exclusion of the washing step, which greatly simplifies the synthesis. For this reason, we decided to test the ability of organic bases, also easily eliminated by combustion, to act as HT precipitants. The use of organic bases, chiefly tetraalkylammonium hydroxides, as reactants in the synthesis of oxide/mixed oxide precursors has been known for over two decades [40,41,42,43]. However, the preparation of HT materials with organic bases is a virtually unexplored field, with a recent paper by Pavel et al. [44] being the rare exception. The authors synthesized an Li-Al layered HT-like compound, for pH-adjustment either using tetramethylammonium hydroxide (TMAOH) or NaOH, and found that the organic base showed an advantage over the inorganic precipitant. With the former, a pure HT phase could be obtained, while co-precipitation with the aid of NaOH yielded Li-Al HT with gibbsite impurity.

The aim of the present work was to (a) explore new alkali-free synthetic routes for the preparation of Mg-Al HT materials by using organic bases as precipitants, (b) compare the physico-chemical and catalytic characteristics of synthesized solids with the properties of HT precipitated with inorganic bases, and (c) investigate the effect of starch biotemplate on the properties of the obtained materials. NaOH and NH_3_aq were chosen as inorganic bases, the former as a reference, the latter as the reactant most commonly used for the preparation of alkali-free HTs, while tetrabutylammonium hydroxide (TBAOH) and choline hydroxide (ChOH) were used in the capacity of organic precipitants. We opted for TBAOH rather than TMAOH because of the exceptional toxicity of the latter, enforcing extreme safety procedures during the handling of the reagent [45]. The attractiveness of ChOH as a precipitating agent is related to its non-toxic nature and biodegradability [46], which make HT synthesis with the use of this reactant particularly benign. The catalytic performance of synthesized solids was assessed in the base catalyzed Baeyer–Villiger oxidation of cyclohexanone to ε-caprolactone using the hydrogen peroxide/nitrile system as an oxidant. The reaction is known to be fostered by a decrease in HT crystal size and increase in the Mg/Al ratio, which are factors sensitive to the synthesis conditions of HT catalysts [33,39,47].

## 2. Materials and Methods

HT materials were synthesized by co-precipitation at pH = 10, using 1 M aqueous solution of Mg and Al nitrates (3:1 molar ratio) as the source of layer-forming cations. The following four bases were used as precipitating agents: 2 M NaOH, 20 wt.% NH_3_aq, 20 wt.% TBAOH and 22.5 wt.% ChOH. In synthesis with NaOH, the carbonate anions were provided by an aqueous solution of Na_2_CO_3_, while in all other syntheses (NH_4_)_2_CO_3_ was used, both acting as reagents in double excess with respect to the stoichiometric demand. We performed a typical synthesis, whereby a solution of Mg and Al nitrates and solution of the precipitating agent, contained in respective burettes, were added dropwise to the beaker with a solution of appropriate carbonate in such a way that the measured pH was kept at a constant level of 10 (±0.05). All inorganic chemicals used for syntheses were of p.a. purity, purchased from Chempur (Piekary Slaskie, Poland). A total of 40 wt.% aqueous TBAOH solution was provided by Sigma-Aldrich (Poznan, Poland), and a 45 wt.% ChOH aqueous solution by Acros Organics (Geel, Belgium). The syntheses were performed at room temperature. The precipitates were washed by 3-fold centrifugation with distilled water, and dried in a drying box. The materials are further referred to as HT-NaOH and HT-NH_3_, HT-TBAOH, and HT-ChOH. Syntheses in the presence of a biotemplate were carried out by dissolving all reagents used for co-precipitation in a gelatinized aqueous solution of starch, prepared by heating in a 0.2 wt.% starch suspension in water at 95 °C for 3 h. Commercial potato starch (PPZ Trzemeszno, Trzemeszno, Poland) was used. The precipitates were washed by 3-fold centrifugation with distilled water, and dried in a drying box at 50 °C. The samples were denoted as HT-NaOH/s, HT-NH_3_/s, HT-TBAOH/s, and HT-ChOH/s.

X-ray diffraction (XRD) patterns were obtained with an X’Pert PRO MPD (PANalytical, Almelo, The Netherlands) diffractometer, with Cu Kα radiation (40 kV, 30 mA), selected using a flat graphite monochromator, and a step size of 0.0334°. A programmable automated divergence slit providing a constant illuminated sample length of 10 mm was used. Crystal sizes (the sizes of coherently scattering domains) of HT solids corresponding to the plate-like crystal thickness and lateral dimension were estimated with the use of the Scherrer equation from the broadening of (003) and (110) reflections, respectively, using the X’Pert High Score software (version 3.0, PANalytical, Almelo, The Netherlands).

A combined thermogravimetric (TG) and differential scanning calorimetry (DSC) analysis was carried out in the flow of air (40 mL/min) with a STA 409 PC LUXX TG/DSC apparatus (Netzsch, Selb, Germany) in the temperature range of 30–1000 °C, at a heating rate of 10 °C/min.

Scanning electron microscopy (SEM) analysis was carried out with the aid of the JEOL JSM-7500F (JEOL, Tokyo, Japan) instrument. SEM images were recorded for the uncoated specimens deposited on 200 mesh copper grids covered with carbon support film.

Inductively coupled plasma optical emission spectrometry (ICP-OES) was used for the determination of Na, Mg and Al. The measurements were carried out by using OPTIMA 2100DV (Perkin-Elmer, Shelton, CT, USA) equipment, after the dissolution of samples in a 1:1 (*v*/*v*) mixture of concentrated HCl and HNO_3_ acids.

The content of C and N in the samples was determined with use of an elemental analyzer Flash SMART (Thermo Fisher Scientific, Bremen, Germany).

Transmission Fourier transform infrared (FTIR) spectra were recorded using a Nicolet 6700 (Thermo Scientific, Madison, WI, USA) spectrometer under atmospheric conditions, in the 4000–400 cm^−1^ range, at a spectral resolution of 2 cm^−1^ for samples prepared as KBr discs.

Quantum mechanical (QM) modeling of ammonia interaction with aquo complexes of Mg^2+^ and Al^3+^ involved geometry optimization and the subsequent determination of the associated energy of the given structure. The considered systems included isolated water and ammonia molecules, Al^3+^ and Mg^2+^ ions with the first coordination sphere composed of water molecules, Al^3+^ and Mg^2+^ ions with the first coordination sphere in which one water molecule was replaced by an ammonia molecule. In the two latter cases, the starting geometries were adopted from the results of the unbiased DFT-MD simulations, carried out for a single metal ion solvated in a bulk solution composed either of pure water (56 molecules) or water with the addition of one ammonia molecule. All QM calculations were performed by using the Gaussian09 software [48] at the MP2/aug-cc-pVTZ level of theory. The DTF-MD simulations were carried out for a single metal ion (either Mg^2+^ or Al^3+^) placed in the cubic simulation box of the edge of ~0.12 nm and solvated with 56 solvent molecules. The interactions within the system were computed within the BP86/def2-SVP potential [49,50]. Simulations were carried with a timestep of 0.25 fs for a duration of 10 ps by using the GROMACS 5 package [51] with ORCA 2.9 [52] applied for DFT calculations.

Catalytic tests of Baeyer–Villiger oxidation of cyclohexanone were carried out for 3 h at 70 °C in thermostated glass batch reactors using Radleys Carousel 6 parallel reaction station (Radleys, Shire Hill, Saffron Walden, Essex, UK) with a magnetic stirrer (500 rpm), in the presence of 0.125 g of catalyst. A total of 12.5 mmol of cyclohexanone, 100 mmol of 30% hydrogen peroxide solution and 200 mmol of acetonitrile were used in the reaction. The reaction mixtures were analyzed by performing gas chromatography using the Thermo Trace GC Ultra instrument (Thermo Electron Corporation, Austin, TX, USA) fitted with a TR-5 capillary column and flame ionization detector. Conversion of cyclohexanone was measured as the percentage of substrate consumed during the reaction. Selectivity to ε-caprolactone was determined as the ratio of the molar quantity of product produced to the molar quantity of converted cyclohexanone, multiplied by 100. Conversion and selectivity data were established as an arithmetic average from 3 parallel experiments for each catalyst (1% error margin). The yield of ε-caprolactone was determined as a product of cyclohexanone conversion and ε-caprolactone selectivity. All reagent and solvents used for catalytic tests were purchased from Merck (Darmstadt, Germany).

## 3. Results and Discussion

### 3.1. Physicochemical Characterization

#### 3.1.1. XRD Analysis

**Figure 2:**
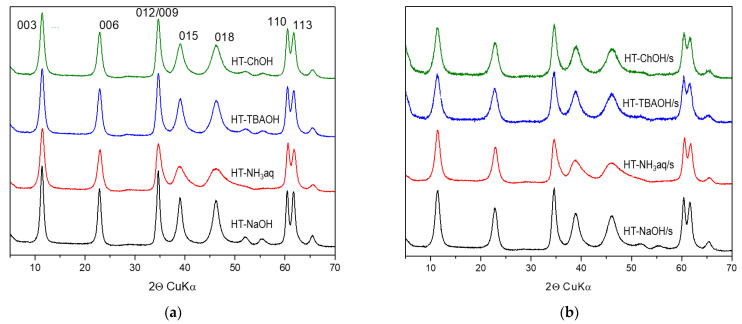
XRD patterns of HT materials obtained with the use of different precipitating bases: (**a**) synthesis in water (**b**) synthesis in aqueous starch solution.

The XRD patterns of HT materials synthesized with different bases in a standard manner and in the presence of starch are gathered in Figure 2a,b, respectively. The data show that all solids possess the structure characteristic of hydrotalcite, free of impurity phases, and the indexing of XRD reflections according to ICSD ref. code 086655 is shown in Figure 2a. The d_003_ interplanar distances listed in Table 1 are in a narrow range of 0.774–0.778 nm, which is consistent with the formation in all cases of a HT structure containing carbonates in the interlayer [2]. The values of d_110_ spacings are also very close to each other, except of those observed for samples prepared with NH_3_aq, which are slightly lower. The d_110_ spacing depends on the mean cation–cation distance in the brucite-like layer. Due to the difference in the ionic radii of Mg^2+^ and Al^3+^ (0.72 Å and 0.54 Å, respectively), d_110_ is sensitive to the degree of Al for Mg substitution [2]. Lowering of the d_110_ value points to the lower Mg/Al ratio in HT materials prepared with ammonia solution. This conclusion is supported by the ICP OES analysis (Table 1). In the case of HT precipitated with NaOH, the TBAOH and ChOH the Mg/Al ratio is very close to the assumed three. On the other hand, in samples precipitated with NH_3_aq the Mg/Al ratio is distinctly lower than the intended value. The result shows that, despite the same constant pH value, the use of ammonia as an alternative to NaOH or organic bases results in the less efficient incorporation of Mg into the HT structure. In addition, the chemical analysis shows that, as expected, traces of sodium are detected only in NaOH precipitated solids (Table 1).

The analysis of diffraction patterns in Figure 2a,b shows that synthesis in the presence of starch results in materials with broader XRD reflections, pointing to their less crystalline character. Indeed, the calculation of crystal sizes in the *c* direction (D_003_) and along the *ab* plane (D_110_) confirms that the use of starch hinders crystal growth and leads to the formation of smaller crystallites (Table 1). The crystal size reduction is least pronounced in the case of the HT-NH_3_/s sample. In both HT series, the samples prepared with NaOH exhibit the highest crystallinity. It is noteworthy that irrespective of the nature of the precipitating base, the decrease in D_003_ is systematically more pronounced than that of the D_110_ parameter. This means that the presence of a biotemplate, aside from reducing the crystal size, modifies its aspect ratio, i.e., the ratio of the crystallite lateral dimension to its thickness (D_110_/D_003_). For materials precipitated in starch solution, the aspect ratio increases, reflecting a more flake-like character of primary crystallites.

#### 3.1.2. QM Analysis of NH_3_ Interaction with Mg^2+^ and Al^3+^ Aquo complexes

The reduced ability of ammonia to effectuate the insertion of magnesium into the HT lattice was reported previously, but no unambiguous explanation of the phenomenon has been offered [15,28,29,30,31,32]. In a couple of works, the Mg deficit was attributed to the pH of the syntheses being too low (between 8 and 9) [15,28]. However, it has been reported that HT materials synthesized at pH 10 or 11 also exhibited a significant deficit of Mg [29,30,32]. Moreover, Tittabut and Trakarnpruk [30], as well as Michalik et al. [32], provided direct evidence that in the case of HT solids synthesized at the same constant pH value with the use of either NaOH or NH_3_aq, the latter precipitant yielded solids with a lower Mg/Al ratio.

In our opinion, the key factor behind the lower than intended Mg content in HT syntheses with NH_3_aq may be related to the ability of Mg^2+^ to form ammine complexes in aqueous solutions of ammonia. According to the literature, hydrated magnesium ion has a weak but measurable tendency to bind ammonia in its coordination sphere [53,54]. In line with the experiment, the theoretical simulation predicted that in an aqueous ammonia solution Mg^2+^ solvated with five water molecules and one ammonia ligand may exist [55]. In view of the rather strong acid character of hydrated Al^3+^ cations, the formation of ammine complexes in aqueous ammonia solutions is not expected to occur. However, in order to directly compare the susceptibility of Mg^2+^ and Al^3+^ aquo complexes to the substitution of H_2_O with NH_3_, we performed QM calculations whose main purpose was to find the difference in energetic effects (Δ*E*) of the ligand-exchange reactions describing the interactions of Al^3+^ and Mg^2+^ ions with water and ammonia:

Al^3+^·(H_2_O)_6_ + NH_3_ → Al^3+^·(H_2_O)_5_·NH_3_ + H_2_O (Δ*E*_1_), (1)

Mg^2+^·(H_2_O)_6_ + NH_3_ → Mg^2+^·(H_2_O)_5_·NH_3_ + H_2_O (Δ*E*_2_). (2)

Equations (1) and (2) are assumed to reflect the realistic process of ligand exchange and provide the base for the energy balance reliance, by providing a quantitative analysis of the favorability of the involved phenomena. The calculated energy balances corresponding to those reactions are equal to Δ*E*_1_ = 38.8 kJ/mol and Δ*E*_2_ = 7.6 kJ/mol. This indicates that replacing one of the water molecules present in the first coordination sphere with the ammonia molecule is energetically favorable neither for Al^3+^ nor for Mg^2+^. However, the corresponding energy change calculated for Al^3+^ is much higher (by more than 30 kJ/mol) than in the case of Mg^2+^. This implies that although water exhibits greater binding affinity in comparison to ammonia for both Al^3+^ and Mg^2+^, the exchange of the H_2_O ligand for NH_3_ is much more likely to occur for Mg^2+^ than for Al^3+^. As mentioned above, in the case of Mg^2+^, the formation of ammine complexes was indeed observed experimentally [53,54]. Such a selective complexation of magnesium is bound to limit the concentration of fully hydrated Mg^2+^ ions available for HT precipitation, and, as a consequence, result in the formation of HT with a lower than intended Mg/Al ratio.

#### 3.1.3. SEM Analysis

**Figure 3:**
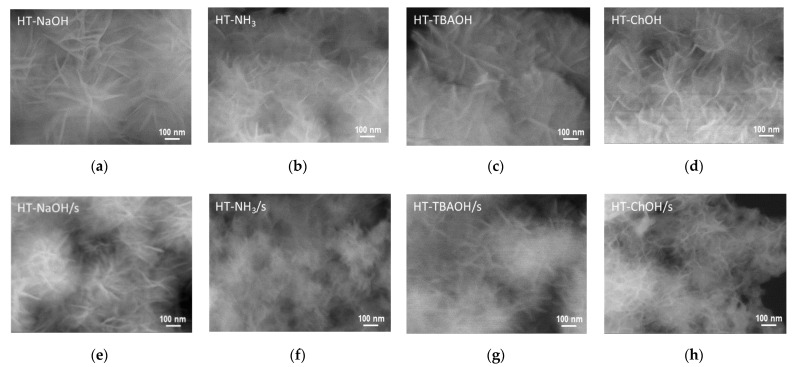
SEM images of HT materials obtained with the use of different precipitating bases in the presence or absence of starch: (**a**) Ht-NaOH; (**b**) Ht-NH_3_; (**c**) Ht-TBAOH; (**d**) Ht-ChOH; (**e**) Ht-NaOH/s; (**f**) Ht-NH_3_/s; (**g**) HT-TBAOH/s; and (**h**) HT-ChOH/s. Magnification × 100,000.

All synthesized HT samples were subjected to an SEM analysis in order to assess the effect of synthesis variables on the materials’ morphology. Representative SEM images are gathered in Figure 3. A comparison of solids obtained with the use of different bases by means of conventional co-precipitation (Figure 3a–d) shows that in all cases the samples exhibited the “sand rose” morphology, usually displayed by Mg-Al HT synthesized at pH = 10 [2,56]. Despite general similarities, it is noticeable that individual flake-like particles precipitated with NaOH are of larger dimensions than those obtained with NH_3_aq, TBAOH or ChOH. All solids obtained with the use of starch (Figure 3e–h) retain the “sand rose” morphology but show a reduction in the platelet grain size with respect to their counterparts prepared without a biotemplate. The result shows that the aqueous solution of starch gel imposes constraints not only on HT primary crystal growth, but also on the formation of HT platelet-like particles, whose size is always much greater than that of the relevant coherently scattering domain. This indicates that individual platelets are not single crystals but constitute packed agglomerates of nanocrystallites.

#### 3.1.4. FTIR Analysis

**Figure 4:**
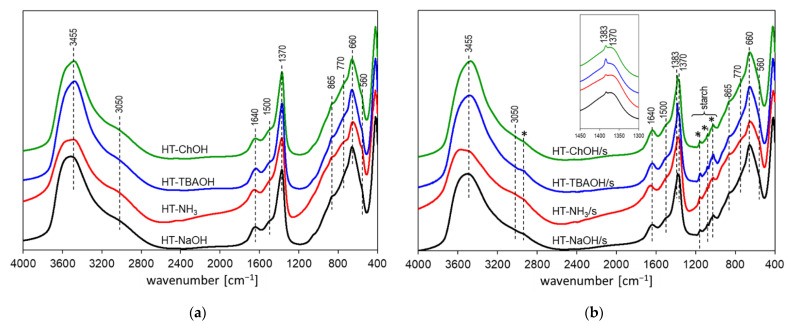
FTIR spectra of HT materials obtained with the use of different precipitating bases: (**a**) synthesis in water (**b**) synthesis in aqueous starch solution (starch bands marked with asterisks).

The FTIR spectra presented in Figure 4 confirm the formation of an HT phase in both series of synthesized solids. Broad complex absorptions between 2800 and 3800 cm^−1^ are a result of the stretching vibrations of OH groups of the brucite-like sheets and of interlayer and/or adsorbed water [57,58]. The maximum absorption of around 3455 cm^−1^ stems from the ν_OH_ stretching modes of hydroxyls in brucite-like layers with significant Al substitution (x > 0.25), and the broad shoulder around 3050 cm^−1^ is due to water hydrogen bonded to the interlayer anions [6,58]. The most intense band in the 1200–1800 cm^−1^ range appears at 1370 cm^−1^ and arises from the asymmetric CO stretch of carbonate anions, while the shoulder at ca. 1500 cm^−1^ originates from the symmetric CO stretching vibration of interlayer bicarbonates. The band at ca. 1640 cm^−1^ is due to bending vibrations in molecular water. In addition, the FTIR spectra of all samples prepared with a starch template reveal the presence of nitrate contamination, manifested as a characteristic sharp band at 1383 cm^−1^ due to symmetric NO stretching in the NO_3_^−^ anion (see insert in Figure 2b) [59,60]. The bands observed in the 400–1200 cm^−1^ range are related to lattice skeleton modes and low frequency vibrations of interlayer anions [57,58,59]. Thus, the maximum at 660 cm^−1^ is due to the in-plane deformation mode of carbonate anions, the shoulders at ca. 770 and 560 cm^−1^ are related to translational modes of OH groups bonded to Al or Mg sites, respectively, while the band at 865 cm^−1^ is due to the out-of-plane deformation mode of carbonate and bicarbonate anions [6]. The set of weak bands in the range of 970–1180 cm^−1^, appearing in the spectra of all members of HT series prepared in the presence of a biotemplate, is due to starch and shows that traces of biopolymer have been retained in the synthesized solids [32]. The presence of nitrate and starch impurities detected in the FTIR spectra was confirmed by the results of the elemental analysis shown in Table 1. The highest amounts of nitrogen and additional carbon were found in the HT-TBAOH/s sample, with other materials showing comparable but slightly lower levels of both elements. The result agrees well with the FTIR spectra, which also show that the presence of nitrate and starch contaminants is most pronounced in HT-TBAOH/s.

#### 3.1.5. Thermal Analysis

**Figure 5:**
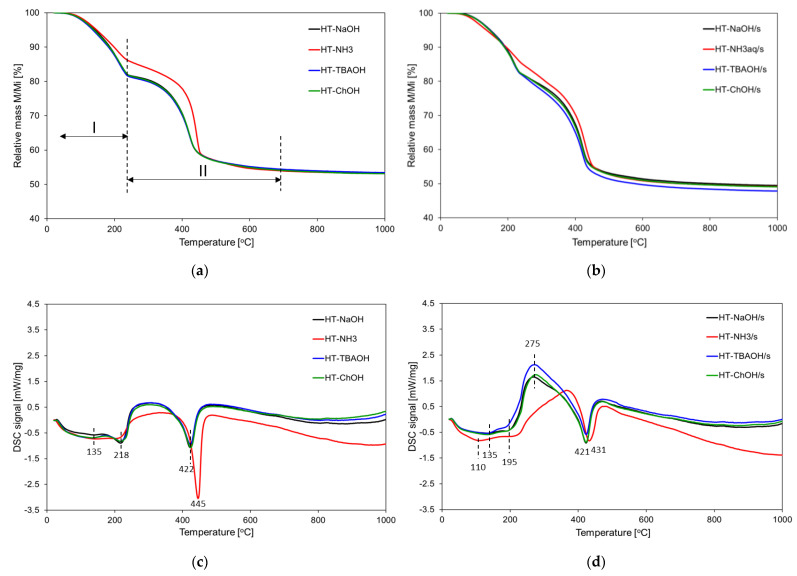
Thermal analysis of HT materials obtained with the use of different precipitating bases: (**a**) TG of samples synthesized in water; (**b**) TG of samples synthesized in aqueous starch solution; (**c**) DSC of samples synthesized in water; (**d**) DSC of samples synthesized in aqueous starch solution.

Results of the thermal analysis of both series of HT materials are presented in Figure 5. All TG traces show that the investigated solids lose weight in two major steps, marked as I and II in Figure 5a, as usually observed for Mg-Al HTs [6,27,61,62,63,64,65]. It has been established that the first step is due to the release of physisorbed and interlayer water, while the second is dominated by the partially overlapping processes of the dehydroxylation of the HT layer and decomposition of interlayer carbonates. In both series, the TG profiles of materials precipitated with NH_3_aq stand out from the rest of the samples whose traces were very similar to each other.

The DSC curves reveal further details of thermal decomposition. In the DSC traces of conventionally precipitated HT series (Figure 3c), three endothermic effects can be distinguished, namely one below ca. 170 °C, related to the release of loosely bound surface water, with a flat maximum at around 135 °C; the next one with a maximum at 218 °C, due to the elimination of interlayer water, and a high temperature effect at above 400 °C, which is associated with concurrent dehydroxylation and decarbonation. In the case of the HT-NH_3_ sample, the latter maximum shifted to a somewhat higher temperature, indicating a slightly better thermal stability of ammonia precipitated HT. For HT-NH_3_/s the shift also exists but is less pronounced. DSC profiles of HT materials synthesized in the presence of starch (Figure 3d) reveal that beside the above described endothermic peaks, with the interlayer dehydration effect shifted to aa slightly lower temperature, a broad exothermic maximum appears in the 250–350 °C range, i.e., lower than expected for combustion of starch in air. It is well resolved for HT-NaOH/s, HT-TBAOH/s and HT-ChOH/s solids, but less so for the HT-NH_3_/s sample. When looking for an explanation of the exothermic effect, one should recall that FTIR analysis, as well as the C and N content determination which reveal that HT samples obtained in the presence of starch contain two types of contaminations including a small amount of nitrates and remnants of starch. It has been reported that starch-nitrate mixtures, upon an increase in temperature, undergo an exothermic pyrolysis in which starch acts as a reductant and nitrate as an oxidant [66]. Therefore, it is proposed that in a biotemplated HT series it is the reaction between the contaminants that is the source of the exothermic phenomenon modifying the typical DSC course of HT decomposition.

### 3.2. Catalytic Testing

**Figure 6:**
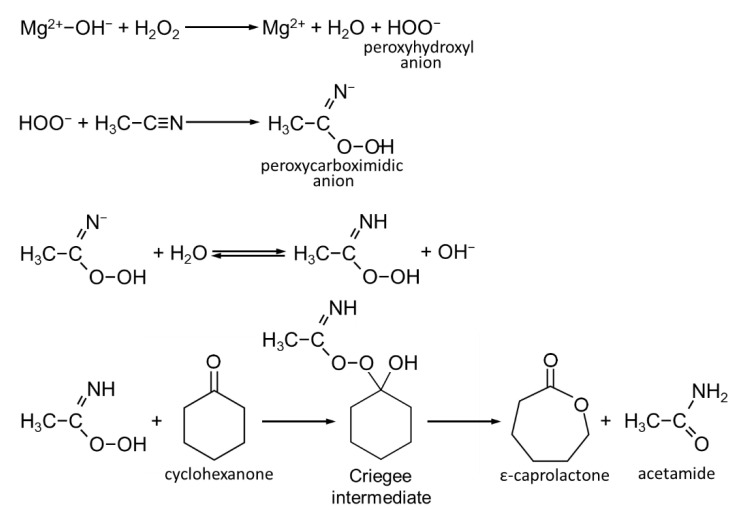
Schematic illustration of reaction steps involved in the oxidation of cyclohexanone to ε-caprolactone with H_2_O_2_/acetonitrile system of Mg-Al HT catalysts. Reaction conditions: 0.125 g of catalyst, 12.5 mmol of cyclohexanone, 100 mmol of 30% H_2_O_2_, 200 mmol of acetonitrile, time = 3 h, temperature = 70 °C, batch reactor.

Synthetic Mg-Al HT materials, as received or calcined, are considered to be highly promising solid base catalysts for a number of liquid phase organic syntheses [67,68,69]. Although solutions of common strong bases such as NaOH or KOH are active in base catalysis, they are also corrosive, harmful, and generate a lot of waste. In contrast, HT solids are non-toxic and environmentally friendly, so their use enables the design of benign catalytic processes. Liquid phase Baeyer–Villiger (B–V) oxidation of cyclohexanone to ε-caprolactone is one of the most studied processes which employs Mg-Al HT as catalysts [33,39,47,70,71,72,73,74,75,76,77]. The reaction product is used for manufacturing polycaprolactone, a biodegradable and biocompatible polymer. In the present work, the liquid phase B–V oxidation of cyclohexanone with hydrogen peroxide was chosen to test the catalytic properties of synthesized HT materials. The reaction was carried out in the presence of acetonitrile, which acts both as a solvent and as a reactant. Its reaction with perhydroxyl anions (HOO−), formed by H_2_O_2_ activation over the Brønsted basic centers at the HT catalyst surface, generates peroxycarboximidic anions, the actual oxidizing intermediate. The ε-caprolactone product is formed in the reaction of peroxycarboximidic acid with cyclohexanone via the so-called Crigee adduct, as illustrated by the reaction scheme shown in Figure 6 [78,79,80]. Our recent works demonstrated that in this reaction the activity of NaOH-precipitated HT catalysts is controlled primarily by the solid’s Mg/Al ratio and crystallinity, which is boosted by a higher Mg content and lower crystal size [33,39,76]. The focus of the current study was to determine how the catalytic performance of solids obtained in the alkali-free syntheses compares with that of catalysts precipitated with NaOH.

**Figure 7:**
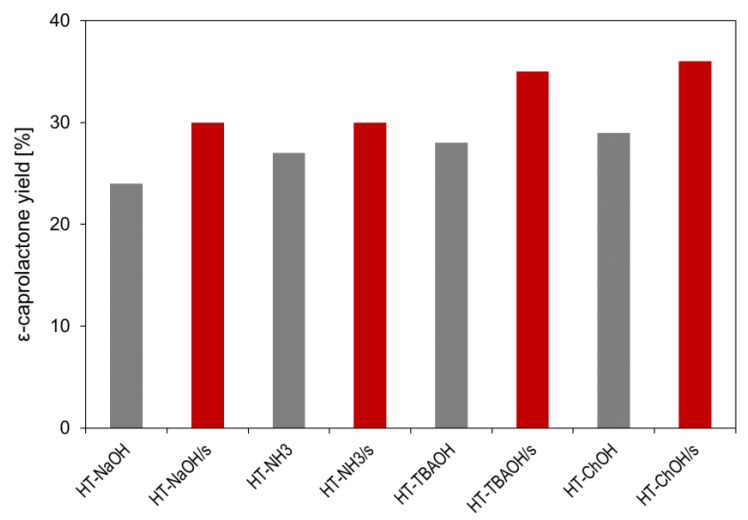
Yield of ε-caprolactone of HT materials obtained with the use of different precipitating bases in the presence or absence of starch. Reaction conditions: 0.125 g of catalyst, 12.5 mmol of cyclohexanone, 100 mmol of 30% H_2_O_2_, 200 mmol of acetonitrile, time = 3 h, temperature = 70 °C, batch reactor.

The catalytic tests showed that on all catalysts, after 3 h of reaction, cyclohexanone was oxidized to ε-caprolactone with high selectivity (90–100%). Longer reaction times resulted in the worsening of selectivity due to the increased consumption of ε-caprolactone in consecutive reactions and/or the increasing contribution of non-selective paths of cyclohexanone conversion. Figure 7 provides a comparison of the obtained ε-caprolactone yields.

The data reveal differences depending on the nature of the precipitating agent and the presence or absence of starch in the HT synthesis medium. Thus, in the case of conventionally prepared HT series all catalysts obtained with precipitants other than NaOH perform better than the HT-NaOH sample used in this study (Figure 6, grey bars). The result may be related to the fact that, according to the data in Table 1, the latter sample is characterized by the highest crystal size, which is unfavorable for catalytic activity. However, the HT-NH_3_ catalyst, with the lowest crystal size, was not the best of the series. Within experimental error, it performed similarly to HT-TBAOH and HT-ChOH. The lower than expected yield of ε-caprolactone over the HT-NH_3_ catalyst may be explained by the lower than intended relative Mg content in ammonia precipitated solids (Table 1). In general, yields of ε-caprolactone observed for catalysts prepared in the absence of starch (24–28%) are at the level previously reported for conventionally synthesized HT with Mg/Al = 3, tested under similar reaction conditions [33,39,73]. In all cases, synthesis in the presence of a biopolymer is beneficial for the catalytic performance, thus confirming the advantage of the fine crystallinity of the catalyst (Figure 6, red bars). It is noteworthy that the yields of ε-caprolactone over HT materials obtained with the use of organic bases, HT-TBAOH/s and HT-ChOH/s, were higher than in the case of catalysts precipitated with inorganic bases. The effect emphasizes drawbacks associated with the use of NaOH or NH_3_aq as precipitants, as the former tends to produce well crystalline solids, while the latter forms complexes which impede the incorporation of magnesium.

We previously demonstrated that in the weakly acidic reaction environment, caused by the presence of H_2_O_2_, some leaching of magnesium from the HT surface occurs, causing a loss of activity upon the reuse of the catalyst [77]. However, the spent catalyst could be easily regenerated by washing with the solution of Mg(OH)_2_. In the present work, the most active HT-ChOH/s sample was subjected to the recycling experiment. The ε-caprolactone yield of the reused catalyst fell to ca. 60% of the value observed in the first run, but after regenerating treatment the catalytic performance was restored to the initial level.

## 4. Conclusions

Organic bases, TBAOH and ChOH, were successfully employed as precipitants in new alkali-free routes of Mg-Al HT synthesis. For comparison, HT solids were also obtained with inorganic bases, NH_3_aq and NaOH. Physicochemical characterization confirmed the formation of well-defined nanocrystalline HT compounds with all employed bases. The HT solids prepared with ammonia differed from all other samples, as they exhibited a lower than intended Mg/Al ratio. The Mg deficit, detrimental to the catalytic activity in base catalyzed reactions, was attributed to the tendency of Mg^2+^, but not Al^3+^, to form ammine complexes; this conclusion was supported by the QM calculations. HT prepared with NaOH showed the highest crystallinity, which was unfavorable for catalytic application. The addition of starch to the synthesis medium provided a way to reduce the crystal size of all HT precipitates. Catalytic tests of Baeyer–Villiger oxidation of cyclohexanone demonstrated that the highest yields of ε-caprolactone were obtained with fine-crystalline HT catalysts prepared with TBAOH and ChOH. In view of this, the use of organic bases should be considered as an attractive option in the synthesis of alkali-free Mg-Al HT materials for application in base catalysis.

## Figures and Tables

**Table 1:** Physico-chemical parameters of synthesized HT solids: interplanar d_003_ and d_110_ distances, crystal sizes in the *c* direction (D_003_) and along *ab* plane (D_110_), D_110_/D_003_ aspect ratio, Mg/Al ratio, Na, C and N content. In brackets degree of crystal size reduction in the presence of starch.

Sample	d_003_ [nm]	d_110_ [nm]	D_003_ [nm]	D_110_ [nm]	D_110_/D_003_	Mg/Al	Na [wt.%]	C [wt.%]	N [wt.%]
HT-NaOH	0.778	0.1532	14.0	20.6	1.47	3.03	0.15	2.28	0
HT-NaOH/s	0.778	0.1532	8.8 (−37%)	15.2 (−26%)	1.72	3.05	0.21	5.18	0.04
HT-NH_3_	0.774	0.1528	9.8	15.1	1.54	2.54	-	2.34	0
HT-NH_3_/s	0.775	0.1528	7.3 (−25%)	13.8 (−9%)	1.89	2.51	-	5.12	0.04
HT-TBAOH	0.776	0.1531	10.9	16.7	1.53	2.97	-	2.37	0
HT-TBAOH/s	0.779	0.1531	6.9 (−37%)	11.8 (−29%)	1.71	3.00	-	6.71	0.11
HT-ChOH	0.777	0.1531	10.4	16.9	1.63	2.93	-	2.39	0
HT-ChOH/s	0.778	0.1531	6.9 (−34%)	13.3 (−21%)	1.93	2.97	-	5.44	0.06

## Data Availability

The data presented in this study are available on request from the corresponding author.
